# Comparative evaluation of the antimicrobial, antioxidant, and cytotoxic properties of essential oils from vetiver, lemongrass, and clove buds with implications for topical application

**DOI:** 10.1371/journal.pone.0335018

**Published:** 2025-10-22

**Authors:** Acharawan Thongmee, Oraphan Wanakhachornkrai, Watchara Chongsa, Patamaporn Sukplang

**Affiliations:** 1 Microbiology Unit, Department of Medical Science, Faculty of Science, Rangsit University, Pathum Thani, Thailand; 2 Physiology Unit, Department of Medical Science, Faculty of Science, Rangsit University, Pathum Thani, Thailand; Università degli Studi di Torino: Universita degli Studi di Torino, ITALY

## Abstract

For many years, essential oils (EOs) have gained attention as natural alternatives to synthetic antimicrobials and antioxidants, although their cytotoxicity remains a major concern for topical use. Leaf EOs from vetiver (*Chrysopogon zizanioides*), lemongrass (*Cymbopogon citratus*), and bud EO from clove (*Syzygium aromaticum*) were tested for their antimicrobial, antioxidant, and cytotoxic activities. Disk diffusion, minimum inhibitory concentration (MIC), and minimum inhibitory concentration (MBC) testing were performed to assess antimicrobial activity against the common skin bacterial pathogens: Methicillin-sensitive *Staphylococcus aureus* (MSSA), Methicillin-resistant *Staphylococcus aureus* (MRSA), *Staphylococcus epidermidis*, and *Pseudomonas aeruginosa*. Clove bud oil exhibited the lowest MIC value of 0.98 μg/mL, whereas vetiver and lemongrass were less effective. In addition, clove bud oil showed the highest antioxidant activity (IC₅₀ value: 3.8 μg/mL for DPPH and 11.3 μg/mL for ABTS). Cytotoxicity on HaCaT cell line showed IC₅₀ for clove, lemongrass and vetiver of 122.14, 123.77 μg/mL and 312.55 μg/mL respectively. The oils of clove bud and vetiver both have antimicrobial activity well below their respective cytotoxic concentrations, indicating a broad margin of safety. However, lemongrass oil required higher concentrations, approaching or exceeding its IC₅₀, particularly against *S. epidermidis*. These results support the potential of clove bud and vetiver oils for safe topical antimicrobial applications, with caution advised for lemongrass.

## 1. Introduction

Essential oils (EOs) have long been known for their biological activities, such as antimicrobial, antioxidant, and anti-inflammatory effects [1,2]. These volatile, plant-derived compounds are widely used in traditional medicine, food preservation, cosmetics, and pharmaceuticals [3]. As global concern over antibiotic resistance and the side effects of synthetic preservatives grows, EOs have emerged as promising natural alternatives [4,5].

Bacteria play two different roles in human health acting as commensals and pathogens. Pathogenic strains, notably methicillin-resistant *Staphylococcus aureus* (MRSA), have become a growing concern in clinical practice, particularly in dermatology [6,7]. The emergence of multi-drug resistant strains raises the need for new antimicrobial compounds [8,9]. EOs have been reported to be potential agents to prevent such bacterial colonization, as they have broad-spectrum antibacterial activity, which is attributed to their membrane disruption, inhibitory effects on metabolic enzymes, and regulation of the expression of microbial genes [10,11].

The EOs of plants are generally made up of a range of compounds, and their composition is influenced by the plant species, the method of extraction, and environmental conditions [12]. Their pharmacological actions of essential oils are primarily driven by their major constituents, including terpenes and their functional derivatives (e.g., phenols such as thymol, aldehydes such as neral). These constituents may interact synergistically to enhance bioactivity or antagonistically to attenuate it [13,14]. Because the activity of EOs depends not only on chemical composition but also on delivery system, their observed biological effects are concentration-dependent and influenced by formulation matrix [15,16].

In addition to their antimicrobial activity, many EOs also offer high antioxidant activity. This is especially important in skincare, where the skin is prone to damage from aging, inflammatory, and environmental-mediated cell damage caused by oxidative stress. Moreover, essential oil from clove bud and vetiver contains eugenol and sesquiterpenes, respectively, which have been reported to be free radical scavengers [17,18].

Nevertheless, the safety of using EOs for treatment is still of concern. High concentrations EOs may have cytotoxic effects on human skin cells such as keratinocytes. A balance between antibacterial and antioxidant activity and cell compatibility is therefore essential for effective topical use. Due to their growing application in various dermatological formulations like creams, lotions, and antimicrobial gels, it is important to determine both the safety and effectiveness of these oils.

This study aims to comprehensively assess the antimicrobial, antioxidant, and cytotoxic properties of three essential oils—vetiver (*Chrysopogon zizaniodes*), lemongrass (*Cymbopogon citratus*), and clove bud (*Syzygium aromaticum*)— against the common skin bacterial pathogens: Methicillin-sensitive *Staphylococcus aureus* (MSSA), Methicillin-resistant *Staphylococcus aureus* (MRSA), *Staphylococcus epidermidis*, and *Pseudomonas aeruginosa*. Cytotoxicity was also evaluated on human HaCaT keratinocytes. The findings aim to inform the development of EO-based formulations that are both effective and safe for skin application.

To date, few studies have directly compared the antimicrobial, antioxidant, and cytotoxic profiles of these three widely used essential oils under standardized conditions. Such comparative analyses are essential to identify oils with optimal efficacy-to-safety ratios for dermal applications.

## 2. Materials and methods

### 2.1. Essential oil

Three essential oils— vetiver oil (*Chrysopogon zizanioides*, Family: Poaceae), lemongrass oil (*Cymbopogon citratus*, Family: Poaceae), and clove bud oil (*Syzygium aromaticum*, Family: Myrtaceae))—were obtained from a natural product retailer (Botanicessence, Bangkok, Thailand). All oils were extracted via hydro-distillation and stored in brown glass vials at 4°C until use.

Gas chromatography (GC) analysis identified the primary constituents of each oil. Vetiver oil was rich in khusimol (38.50%), β-vetivenene (22.75%), vetiverol (15.20%), α-vetivone (6.95%), and β-vetivone (5.30%). Lemongrass oil contained geranial (47.46%), neral (33.34%), geraniol (5.30%), citronellal (2.53%), myrcene (1.10%), linalool (1.07%), geranyl acetate (0.85%), and limonene (0.35%). Clove bud oil consisted predominantly of eugenol (80.50%), followed by eugenyl acetate (9.77%), β-caryophyllene (7.26%), α-humulene (0.81%), ledene (0.57%), caryophyllene oxide (0.54%), and cyclohexane (0.33%).

### 2.2. Bacterial strains

The bacterial strains used in this study were *Staphylococcus aureus* ATCC® 25923™ (MSSA), *Staphylococcus aureus* ATCC® 43300™ (MRSA), *Staphylococcus epidermidis* ATCC® 12228™, and *Pseudomonas aeruginosa* ATCC® 27853™, which are commonly associated with skin infections and widely used as reference strains for antimicrobial testing. All strains were revived from glycerol stocks and cultured in Tryptic Soy Broth (TSB) prior to use.

### 2.3. Antimicrobial activity of essential oils

#### 2.3.1. Disk diffusion method.

Each bacterial strain was cultured in TSB at 37°C for 18–24 hours. The cultures were standardized to a 0.5 McFarland turbidity standard (~10⁸ CFU/mL). For the disk diffusion assay, 100 µL of each bacterial suspension was uniformly spread on Tryptic Soy Agar (TSA) plates using a sterile swab.

Sterile 6 mm paper disks were impregnated with 10 µL of essential oil and placed on the inoculated agar surface. Plates were incubated at 37°C for 24 hours. Zones of inhibition were measured in millimeters. Each test was performed in triplicate.

#### 2.3.2. Minimum inhibitory concentration (MIC) and minimum bactericidal concentration (MBC).

MIC and MBC values were determined using the broth microdilution method. Two-fold serial dilutions of each EO were prepared in TSB in 96-well microtiter plates. Each well received 100 µL of EO dilution and 100 µL of bacterial suspension (~10⁶ CFU/mL).

Positive controls contained bacteria without EOs, and sterility controls contained broth only. Plates were incubated at 37°C for 18–24 hours. MIC was defined as the lowest EO concentration inhibiting visible bacterial growth.

For MBC determination, 10 µL from wells showing no growth were subcultured onto TSA and incubated at 37°C for 24 hours. MBC was the lowest concentration with no colony growth. All tests were conducted in triplicate.

### 2.4. Antioxidant assay

To comprehensively evaluate antioxidant capacity, the DPPH (1,1-diphenyl-2-picrylhydrazyl) and ABTS [2,2’-azino-bis(3-ethylbenzothiazoline-6-sulfonic acid)] radical scavenging assays were conducted. These methods evaluate different antioxidant mechanisms, thereby ensuring a more robust and reliable analysis [19,20]. Antioxidant activity was quantified using the IC_50_ value, defined as the concentration of the sample required to scavenge 50% of the DPPH or ABTS radicals. All antioxidant experiments were performed in triplicate, and results are expressed as mean ± SEM.

#### 2.4.1. DPPH radical scavenging activity.

The DPPH assay was performed as previously described by Benkhaira et.al [21], with slight modifications. A 0.1 mM DPPH solution was prepared in methanol. Essential oils and Trolox (standard) were diluted to desired concentrations in methanol.

In a 96-well plate, 100 µL of sample or control solution was mixed with 100 µL of DPPH. The mixture was incubated in the dark at room temperature for 30 minutes. Absorbance was measured at 517 nm using a microplate reader. Radical scavenging activity (%RSA) was calculated using the formula:


$$\%RSA\;=\frac{\left(\;A_{Control}\;-A_{sample}\;\right)}{A_{Control}\;}\times 100$$

#### 2.4.2. ABTS radical scavenging activity.

Following the procedure described by Re et.al [20], the ABTS radical cation (ABTS•⁺) was generated by mixing ABTS with potassium persulfate and allowing the reaction to proceed in the dark for 12–16 hours. The resulting ABTS• ⁺ solution was diluted with methanol to an absorbance of 0.7 at 734 nm.

In a 96-well plate, 100 µL of EO dilution or Trolox was mixed with 100 µL of ABTS• ⁺ solution and incubated in the dark at room temperature for 15 minutes. Absorbance was recorded at 734 nm. %RSA was calculated as described above.

### 2.5. Cytotoxicity assay

The cytotoxicity of the essential oils was assessed on human HaCaT keratinocytes using the MTT assay. HaCaT cells, sourced from Cell Lines Service (Heidelberg, Germany), were cultured in Dulbecco’s Modified Eagle Medium (DMEM) supplemented with 10% fetal bovine serum, 1% penicillin-streptomycin, and 1% GlutaMAX. The HaCaT keratinocyte cell line was chosen to represent human skin cells in order to evaluate possible cytotoxic effects that might occur with topical application.

Cells were seeded in 96-well plates at 1 × 10⁴ cells/well and allowed to adhere for 24 hours. Subsequently, the cells were treated with six different concentrations of EOs (1, 10, 100, 200, 400 and 500 µg/mL), and the plates were further incubated for 24 hours. After 24-hour incubation, the cells were washed with PBS and incubated with MTT solution for 3 hours. After discarding the supernatant, DMSO was added to solubilize formazan crystals. Absorbance was measured at 570 nm.

Cell viability was calculated relative to untreated control wells. The IC₅₀ value, the concentration at which each essential oil exerted half of its maximal inhibitory effect, was determined using the Quest Graph™ IC_50_ Calculator [22]. All experiments were performed in triplicate and included appropriate positive and negative controls.

### 2.6. Statistical analysis

Data are expressed as mean ± SEM from at least three independent experiments. Differences between the control and Trolox in antioxidant assays were analyzed using the Student’s t-test. Comparisons among essential oil groups across concentrations and for IC₅₀ values were assessed by one-way ANOVA followed by Tukey’s post hoc test. Statistical significance was accepted at **p* *< 0.05.

## 3. Results

### 3.1. Antimicrobial activity

The antimicrobial properties of vetiver, lemongrass, and clove bud EOs against the tested bacterial strains are presented in Tables 1 and 2.

**Table 1:** Zones of inhibition of essential oils against tested bacterial strains.

Essential oils	Zone of inhibition (mm ± SD)			
	*S. aureus* (MSSA)	*S. aureus* (MRSA)	*S. epidermidis*	*P. aeruginosa*
Vetiver	10.3 ± 0.3	10.3 ± 0.6	15.3 ± 0.4	No
Lemongrass	34.3 ± 0.6	30.3 ± 0.5	37.7 ± 0.3	No
Clove bud	19.3 ± 0.4	15.3 ± 0.3	19.3 ± 0.6	7.3 ± 0.6

No indicates no inhibition.

**Table 2:** Minimum inhibitory concentration (MIC) and minimum bactericidal concentration (MBC) of essential oils against tested bacterial strains.

Essential oils	*S. aureus* (MSSA)		*S. aureus* (MRSA)		*S. epidermidis*		*P. aeruginosa*	
	MIC	MBC	MIC	MBC	MIC	MBC	MIC	MBC
	(μg/mL)	(μg/mL)	(μg/mL)	(μg/mL)	(μg/mL)	(μg/mL)	(μg/mL)	(μg/mL)
Vetiver	3.87	15.47	7.73	15.47	7.73	123.75	No	No
Lemongrass	27.81	55.63	27.81	55.63	222.5	445	No	No
Clove bud	1.95	3.91	3.91	3.91	0.98	1.95	3.91	62.5

No indicates no inhibition.

In the disk diffusion assay, lemongrass oil exhibited the largest zones of inhibition against all three Gram-positive strains, with diameters of 34.3 ± 0.6 mm (MSSA), 30.3 ± 0.5 mm (MRSA), and 37.7 ± 0.3 mm (*S. epidermidis*). Clove bud oil produced moderate inhibition zones ranging from 15.3 ± 0.3 mm to 19.3 ± 0.6 mm across the same strains. Vetiver oil showed weaker activity, with zones of 10.3 ± 0.3 mm (MSSA and MRSA) and 15.3 ± 0.4 mm (*S. epidermidis*). Notably, only clove bud oil exhibited activity against *P. aeruginosa*, with a zone of 7.3 ± 0.6 mm, while vetiver and lemongrass oils showed no inhibition.

MIC and MBC values confirmed the superior antimicrobial efficacy of clove bud oil. It displayed the lowest MIC values, ranging from 0.98 to 3.91 µg/mL, and MBC values from 1.95 to 62.5 µg/mL. Vetiver oil showed moderate MICs (3.87–7.73 µg/mL) against Gram-positive strains but was ineffective against *P. aeruginosa*. Lemongrass oil demonstrated strong activity against MSSA and MRSA (MIC = 27.81 µg/mL) but required much higher concentrations to inhibit *S. epidermidis* (MIC = 222.5 µg/mL), and it showed no activity against *P. aeruginosa*.

### 3.2. Antioxidant assay

All essential oils tested showed dose-dependent radical scavenging activity that increased with concentration.

#### 3.2.1. DPPH assay.

The Clove bud EO exhibited the strongest antioxidant activity, achieving a maximum radical scavenging activity (%RSA) of 88.44% ± 0.39% at 5 mg/mL. Notably, it maintained high RSA at lower concentrations, such as 0.078 mg/mL (86.80% ± 0.59%) and 0.039 mg/mL (84.30% ± 0.65%), with an IC₅₀ value of 3.8 μg/mL. Vetiver EO followed, with a peak RSA of 86.33% ± 0.39% at 5 mg/mL and an IC₅₀ of 789.80 μg/mL. Lemongrass EO exhibited the weakest scavenging activity, with a maximum RSA of 57.69% ± 2.01% and an IC₅₀ of 1906.20 μg/mL (S1 Table, Table 3, S1 Fig).

**Table 3:** IC_50_ values from DPPH and ABTS assays (mean±SEM).

Treatment	IC_50_ (μg/mL)	
**DPPH Assay**	**ATBS Assay**
Trolox	6.49 ± 0.47	8.23 ± 0.41
Vetiver EO (VET)	790.37 ± 13.64 ^a,c,d^	265.43 ± 11.20 ^a,c,d^
Lemongrass EO (LG)	1948.60 ± 53.53 ^a,b,d^	1733.90 ± 50.95 ^a,b,d^
Clove bud EO (CB)	8.70 ± 2.26 ^a,b,c^	3.54 ± 0.20 ^a,b,c^

Data are expressed as mean ± SEM of triplicate individual experiments.

^a^Significantly different when compared with Trolox group

^b,c,d^Significantly different from VET, LG and CB group respectively

#### 3.2.2. ABTS assay.

The ABTS assay confirmed the trends observed in the DPPH assay. Clove bud EO achieved a maximum RSA of 95.02% ± 0.27% at 0.078 mg/mL and maintained high RSA values (>94%) across all tested concentrations (5.0 to 0.039 mg/mL), with an IC₅₀ of 11.3 μg/mL. Vetiver EO peaked at 94.90% ± 0.26% at 1.25 mg/mL, with an IC₅₀ of 263.20 μg/mL. Lemongrass EO showed its highest RSA (92.11% ± 0.19%) at 5 mg/mL and had an IC₅₀ of 1548.60 μg/mL (S2 Table, Table 3, S2 Fig).

Consistent with the supplementary RSA concentration series, clove bud oil showed the strongest radical scavenging with IC₅₀ values of 8.70 ± 2.26 μg/mL (DPPH) and 3.54 ± 0.20 μg/mL (ABTS), followed by vetiver, whereas lemongrass exhibited the weakest antioxidant capacity (Table 3).

### 3.3. Cytotoxicity assay

**Fig 1:**
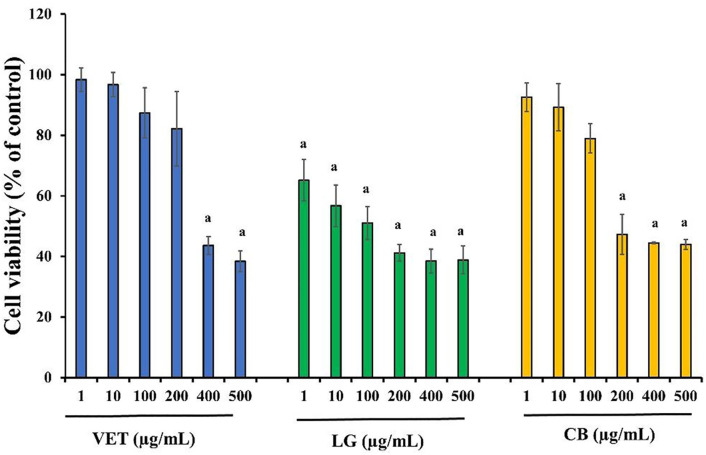
Effect of essential oils on the viability of HaCaT cell line. ^a^ *p *< 0.05 compare with control.

As the concentration of each essential oil increased, the viability of HaCaT keratinocytes decreased accordingly (Table 4, Fig 1).

**Table 4:** Percent cell viability (relative to control) of HaCaT keratinocytes after 24-hour exposure to different concentrations of essential oil.

	% Cell viability (relative to control) (Mean±SEM)		
	Vetiver oil (VET)	Lemongrass oil (LG)	Clove bud oil (CB)
1 µg/mL	98.36 ± 3.86	65.14 ± 6.89 ^a^	92.57 ± 4.72
10 µg/mL	96.68 ± 3.99	56.75 ± 6.86 ^a^	89.23 ± 7.84
100 µg/mL	87.38 ± 8.34	51.08 ± 5.40 ^a^	78.92 ± 4.85
200 µg/mL	82.1 ± 12.30	41.15 ± 2.75 ^a^	47.29 ± 6.61 ^a^
400 µg/mL	43.62 ± 2.97^a^	38.46 ± 3.97 ^a^	44.46 ± 0.29 ^a^
500 µg/mL	38.38 ± 3.37 ^a^	38.38 ± 4.58 ^a^	43.95 ± 1.63 ^a^
**IC**_**50**_	**368.06 ± 26.71**	**109.99 ± 10.13** ^**a**^	**122.49 ± 12.45** ^**a**^

**p* *< 0.05 compare with control

^a^Significantly lower than control

At 100 μg/mL, clove bud EO reduced cell viability to 78.92%, with a more pronounced effect observed at 200 μg/mL (47.29%). Vetiver and lemongrass EOs also showed decreased viability at 200 μg/mL, with values of 82.10% and 41.15%, respectively. At 400–500 μg/mL, all EOs significantly reduced cell viability to below 45%. The IC₅₀ values on HaCaT cells were 122.49 ± 12.45 μg/mL for clove bud oil, 109.99 ± 10.13 μg/mL for lemongrass oil, and 368.06 ± 26.71 μg/mL for vetiver oil, indicating that vetiver was the least cytotoxic among the three.

## 4. Discussion

Our results demonstrate that the essential oils (EOs) of vetiver, lemongrass, and clove bud possess notable antimicrobial, antioxidant, and cytotoxic activities. These findings are in line with previous research that highlights the broad-spectrum biological properties of plant-derived EOs [1,2]. In particular, the significant antibacterial effects observed in our study support the potential application of these oils as natural therapeutic agents for managing skin infections [5].

All three essential oils demonstrated clear antibacterial activity against the Gram-positive strains tested, including methicillin-sensitive *Staphylococcus aureus* (MSSA), methicillin-resistant *S. aureus* (MRSA), and *Staphylococcus epidermidis*. These findings align with previous reports suggesting that Gram-positive bacteria tend to be more susceptible to essential oils than Gram-negative species, largely due to their simpler cell wall structures, which allow easier penetration of lipophilic compounds such as terpenes and phenols [10,13]. Among the oils tested, clove bud oil exhibited the strongest antibacterial effect, with a remarkably low MIC of 0.98 μg/mL, confirming its potent bactericidal activity [13]. This high efficacy is primarily attributed to its rich eugenol content, a bioactive compound known to disrupt bacterial membranes, inhibit key enzymes, and induce oxidative stress within microbial cells [23,24].

Lemongrass oil produced the largest zones of inhibition in the disk diffusion assays, aligning with earlier studies that link its antimicrobial effects to citral, its primary component, known for disrupting bacterial membranes and interfering with quorum sensing pathways [14,25]. However, its relatively high MIC values, especially against *Staphylococcus epidermidis*, suggest that the oil’s potency in agar-based diffusion tests may not translate as effectively in broth-based conditions.

The apparent discrepancy for lemongrass oil, which produced large inhibition zones on agar yet required high MICs in broth, is consistent with method-dependent behavior of volatile and hydrophobic constituents such as citral. In agar diffusion, both lateral diffusion in the hydrated matrix and vapor-phase effects can contribute to visible zones, whereas in broth microdilution, solubility, partitioning, and emulsification constraints limit the free concentration at the cell interface. These differences between vapor or surface assays and liquid microdilution have been reported for essential oils, and they explain why agar potency may not fully translate to broth conditions for *S. epidermidis* [14,25,26].

In contrast, vetiver oil demonstrated only moderate activity against Gram-positive bacteria and showed no inhibitory effect against *Pseudomonas aeruginosa*, a notoriously resistant Gram-negative species with a robust outer membrane and multiple defense mechanisms [8]. Interestingly, clove bud oil was the only one to show measurable activity against *P. aeruginosa*, though at higher concentrations. This is particularly significant given the innate resistance of Gram-negative bacteria and further supports previous findings highlighting clove oil’s broad-spectrum antimicrobial potential [10].

The variation in antimicrobial activity observed among the different oils and bacterial species is consistent with earlier reports suggesting that the efficacy of an essential oil depends not only on its chemical composition, but also on the specific bacterial strain and the testing method used [26]. Although vetiver oil was less active than clove oil, it still showed moderate activity against Gram positive species. This effect is likely related to its sesquiterpene components, such as khusimol, which have been shown to trigger membrane stress responses and programmed cell death in bacterial cells [27]. The close alignment between the MIC and MBC values for clove bud oil also suggests a primarily bactericidal mode of action, which could offer an advantage in minimizing the development of bacterial resistance [8].

From an application perspective, clove bud oil may serve as a multifunctional ingredient for topical use, offering both antimicrobial and antioxidant benefits. However, its cytotoxicity at higher concentrations highlights the need for careful formulation strategies, such as nanoencapsulation or dilution with non-toxic carriers. While vetiver oil exhibited the lowest antimicrobial activity among the oils tested, it was also the least cytotoxic, suggesting that it may be better suited for products designed for sensitive skin. These findings are particularly relevant for the development of dermatological formulations targeting conditions like acne, folliculitis, or superficial bacterial infections, where effective local antimicrobial action with minimal irritation is essential.

Given the broad antimicrobial and antioxidant performance of clove bud oil but its higher cytotoxicity at elevated doses, combining clove bud with the less cytotoxic vetiver oil may enable lower clove concentrations while preserving antibacterial efficacy and improving tolerability for sensitive skin. The synergistic effects in EO blend strategy has been previously reported [11].

The results therefore provide applicable information for the development of EO-based skin products. The above findings are especially important for dermatological formulations destined for the treatment of conditions such as acne, folliculitis, or superficial bacterial infections, where a topical antimicrobial action against the pathogen is required, but irritation of the skin is to be held to a minimum.

With regard to antioxidant efficacy, clove bud oil demonstrated the broadest radical scavenging capacity in both DPPH and ABTS assays. This effect is widely linked to the well-established antioxidant properties of eugenol [17]. The phenolic hydroxyl group of eugenol is believed to neutralize free radicals through electron or hydrogen donation. Vetiver oil also showed some antioxidant activity, likely resulting from its sesquiterpene constituents such as β-vetivenene and khusimol [18], making it a promising candidate for developing formulations aimed at treating oxidative skin damage [28].

Dose-dependent results were confirmed for all the three oils in cytotoxicity. Among them, vetiver oil exhibited the least cytotoxicity (IC₅₀ = 312.55 µg/mL), and clove and lemongrass oils presented with similar IC₅₀ values (122.14 and 123.77 µg/mL, respectively). Importantly, clove bud oil’s MICs were significantly lower than its IC₅₀, indicating a broad therapeutic index. In comparison, lemongrass oil, except for effects near or above its IC₅₀, had no statistically significant inhibition against *S. epidermidis*, giving reasons against cutaneous use. These findings are consistent with prior research demonstrating that clove oil and its major component eugenol are cytotoxic dose-dependently [23,29].

To relate efficacy to cytotoxicity, we compared MICs with HaCaT IC₅₀ values (S3 Table). Clove bud oil showed favorable indices across Gram-positive strains, for example IC₅₀/MIC ≈ 125 for *S. epidermidis* (122.49/0.98), ≈ 63 for MSSA (122.49/1.95), and ≈ 31 for MRSA and *P. aeruginosa* (122.49/3.91). Vetiver exhibited indices of ≈ 95 for MSSA (368.06/3.87) and ≈ 48 for MRSA and *S. epidermidis* (368.06/7.73). In contrast, lemongrass had narrow margins for MSSA and MRSA (≈ 4; 109.99/27.81) and an unfavorable ratio for *S. epidermidis* (≈ 0.49; 109.99/222.5), consistent with cautious use against this species. At sub-cytotoxic levels that are relevant to topical formulations, clove and vetiver maintained activity while HaCaT viability remained comparatively high at 10–100 μg/mL, whereas lemongrass reduced viability more sharply at equivalent doses. This type of comparative analysis between antimicrobial potency and cytotoxic thresholds has been employed previously to estimate therapeutic indices for essential oils and their components [23,30,31]. Such evaluations support the relevance of therapeutic window calculations for guiding formulation safety and highlight the potential of clove and vetiver oils for topical applications where cytotoxicity must be minimized [32].

Despite eugenol is classified as a Generally Recognized as Safe (GRAS) compound, it might be metabolically activated as quinone methide and decrease cellular glutathione levels, leading to apoptosis or necrosis [23]. However, the cytotoxic concentrations determined in this work are within those found in commercial personal care products (CPG’s), indicating that if formulated and dosed properly, clove bud oil can be topically applied while keeping in mind its availability and low cost [29,33].

Although these results offer convincing in vitro evidence to support that the selected EOs have superior relative antimicrobial activity and/or safety profiles, limitations exist in this study. These findings were obtained based on cell-based and microbial assays, which do not comprehensively reflect the skin physiology and pharmacokinetics of topical oil use. Furthermore, while the antimicrobial feature of oils was found to be effective, we did not use a carrier, which would likely impact performance in practice. Further investigations on skin models or formulation optimization are needed to confirm these results.

In conclusion, clove bud oil showed the most optimal combination of potent antimicrobial and antioxidant activities and acceptable cytotoxicity and thus could be considered as the most hopeful candidate for incorporation in the dermal formulations. Instead of using pure vetiver root oil, it can be replaced with a safer and milder choice as vetiver oil. However, the modest therapeutic window of lemongrass oil on some strains indicates that formulation should be approached with care. The data presented here emphasize the value of evaluating efficacy and safety for the potential medicinal or cosmetic use of EOs and provide a scientific basis that may help to guide the development of safe and effective EO-based skin care products.

As these results are based solely on in vitro assays, they may not fully capture the complexity of skin physiology, absorption, and metabolism. Futher in vivo and formulation studies are needed to confirm safety and efficacy before clinical application.

## 5. Conclusions

This study highlights the diverse biological potential of clove bud, lemongrass, and vetiver essential oils, particularly in the context of skin-related applications. Clove bud oil stood out for its strong antimicrobial and antioxidant activities, though its cytotoxicity at higher concentrations calls for careful formulation. Lemongrass oil showed pronounced antibacterial effects in diffusion-based testing, while vetiver oil, though less potent, demonstrated low toxicity and moderate antioxidant action. Taken together, these findings support the potential use of these natural oils as active ingredients in dermatological products, especially where gentle but effective antimicrobial and antioxidant effects are desired.

## Supporting information

S1 Fig Effect of essential oils on percent radical scavenging activity in the DPPH assay. ^a^ *p* < 0.05 compare with control, ^b^ *p* < 0.05 compare with Trolox.
(PDF)

S2 Fig Effect of essential oils on percent radical scavenging activity in the ABTS assay. ^a^ *p *< 0.05 compare with control, ^b^ *p *< 0.05 compare with Trolox.
(PDF)

S1 Table Percent radical scavenging activity (%RSA) of Trolox, vetiver, lemongrass, and clove bud essential oils at various concentrations, as determined by the DPPH assay. (PDF)

S2 Table Percent radical scavenging activity (%RSA) of Trolox, vetiver, lemongrass, and clove bud essential oils at various concentrations, as determined by the ABTS assay. (PDF)

S3 Table Selectivity index (IC₅₀/MIC ratios) of clove bud, lemongrass, and vetiver essential oils against tested bacterial strains. (PDF)
