# Supplementary material for: Comparative evaluation of the antimicrobial, antioxidant, and cytotoxic properties of essential oils from vetiver, lemongrass, and clove buds with implications for topical application
Source: PLoS One. 2025 Oct 22;20(10):e0335018. doi: 10.1371/journal.pone.0335018 (PMC12543172; doi:10.1371/journal.pone.0335018)
Supplement: S1 Fig — a p < 0.05 compare with control, b p < 0.05 compare with Trolox. (PDF) [file pone.0335018.s001.pdf]

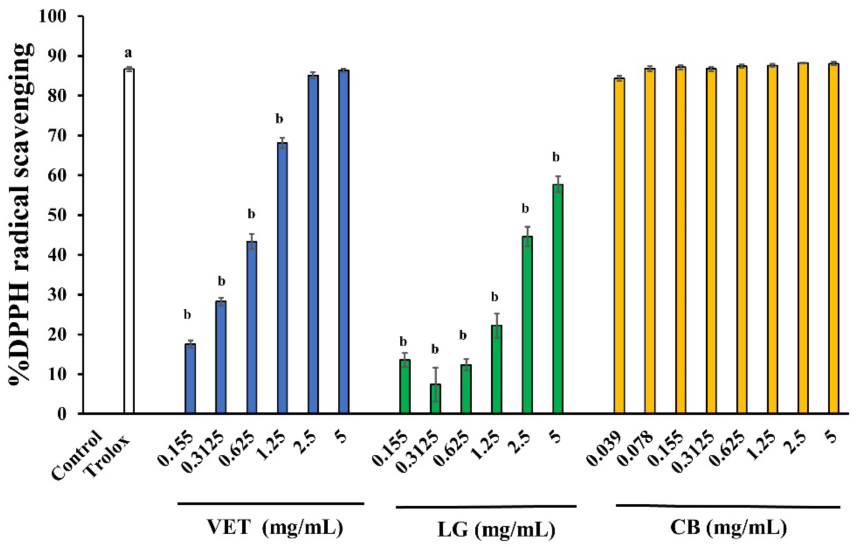

S1 Fig. Effect of essential oils on percent radical scavenging activity in the DPPH assay
[a] $p < 0.05$ compare with control, [b] $P<0.05$ compare with Trolox
