# Supplementary material for: Comparative evaluation of the antimicrobial, antioxidant, and cytotoxic properties of essential oils from vetiver, lemongrass, and clove buds with implications for topical application
Source: PLoS One. 2025 Oct 22;20(10):e0335018. doi: 10.1371/journal.pone.0335018 (PMC12543172; doi:10.1371/journal.pone.0335018)
Supplement: S1 Table — (PDF) [file pone.0335018.s003.pdf]

S1 Table. Percent radical scavenging activity (%RSA) of Trolox, vetiver, lemongrass, and clove bud essential oils at various concentrations, as determined by the DPPH assay

	**Concentration**	**DPPH**<br>**% Radical Scavenging Activity (RSA) ± SD**
Trolox	12.5 μg/mL	86.70 ± 0.57 [a]
Vetiver oil (VET)	5 mg/mL	86.33 ± 0.39
	2.5 mg/mL	85.08 ± 0.77
	1.25 mg/mL	68.14 ± 1.31
	0.625 mg/mL	43.34 ± 1.89 [b]
	0.3125 mg/mL	28.27 ± 0.97 [b]
	0.155 mg/mL	17.56 ± 0.09 [b]
Lemongrass oil (LG)	5 mg/mL	57.69 ± 2.01 [b]
	2.5 mg/mL	44.59 ± 2.49 [b]
	1.25 mg/mL	22.24 ± 3.05 [b]
	0.625 mg/mL	12.31 ± 1.42 [b]
	0.3125 mg/mL	7.42 ± 4.27 [b]
	0.155 mg/mL	13.55 ± 1.77 [b]
Clove bud oil (CB)	5 mg/mL	88.44 ± 0.39
	2.5 mg/mL	88.2 ± 0.18
	1.25 mg/mL	87.58 ± 0.39
	0.625 mg/mL	87.42 ± 0.39
	0.3125 mg/mL	86.74 ± 0.54
	0.155 mg/mL	87.16 ± 0.59
	0.078 mg/mL	86.8 ± 0.59
	0.039 mg/mL	84.3 ± 0.65
