# Supplementary material for: Comparative evaluation of the antimicrobial, antioxidant, and cytotoxic properties of essential oils from vetiver, lemongrass, and clove buds with implications for topical application
Source: PLoS One. 2025 Oct 22;20(10):e0335018. doi: 10.1371/journal.pone.0335018 (PMC12543172; doi:10.1371/journal.pone.0335018)
Supplement: S2 Table — (PDF) [file pone.0335018.s004.pdf]

S2 Table. Percent radical scavenging activity (%RSA) of Trolox, vetiver, lemongrass, and clove bud essential oils at various concentrations, as determined by the ABTS assay

	**Concentration**	**ABTS<br>% Radical Scavenging Activity (RSA) ± SD**
Trolox	12.5 µg/mL	67.46 ± 5.24 [a]
Vetiver oil (VET)	5 mg/mL	94.60 ± 0.27
	2.5 mg/mL	94.48 ± 0.44
	1.25 mg/mL	94.90 ± 0.26
	0.625 mg/mL	87.28 ± 1.80
	0.3125 mg/mL	67.91 ± 2.55 [b]
	0.155 mg/mL	40.74 ± 0.54 [b]
Lemongrass oil (LG)	5 mg/mL	92.11 ± 0.19 [b]
	2.5 mg/mL	81.68 ± 2.22 [b]
	1.25 mg/mL	65.87 ± 2.16 [b]
	0.625 mg/mL	54.57 ± 1.65
	0.3125 mg/mL	45.45 ± 1.33 [b]
	0.155 mg/mL	42.36 ± 0.30 [b]
Clove bud oil (CB)	5 mg/mL	94.87 ± 0.32 [b]
	2.5 mg/mL	94.99 ± 0.19 [b]
	1.25 mg/mL	94.90 ± 0.29 [b]
	0.625 mg/mL	94.66 ± 0.37 [b]
	0.3125 mg/mL	94.81 ± 0.23 [b]
	0.155 mg/mL	94.87 ± 0.09 [b]
	0.078 mg/mL	95.02 ± 0.27 [b]
	0.039 mg/mL	94.39 ± 0.21 [b]
