# Supplementary material for: Comparative evaluation of the antimicrobial, antioxidant, and cytotoxic properties of essential oils from vetiver, lemongrass, and clove buds with implications for topical application
Source: PLoS One. 2025 Oct 22;20(10):e0335018. doi: 10.1371/journal.pone.0335018 (PMC12543172; doi:10.1371/journal.pone.0335018)
Supplement: S3 Table — (PDF) [file pone.0335018.s005.pdf]

S3 Table. Selectivity index (IC_{50}/MIC ratios) of clove bud, lemongrass, and vetiver essential oils against tested bacterial strains.

Essential Oil	HaCaT IC_{50} (µg/mL)	Species	MIC (µg/mL)	IC50/MIC
Clove bud EO	122.49	MRSA	3.91	31.33
Clove bud EO	122.49	MSSA	1.95	62.82
Clove bud EO	122.49	*P. aeruginosa*	3.91	31.33
Clove bud EO	122.49	*S. epidermidis*	0.98	124.99
Lemongrass EO	109.99	MRSA	27.81	3.96
Lemongrass EO	109.99	MSSA	27.81	3.96
Lemongrass EO	109.99	*S. epidermidis*	222.5	0.49
Vetiver EO	368.06	MRSA	7.73	47.61
Vetiver EO	368.06	MSSA	3.87	95.11
Vetiver EO	368.06	*S. epidermidis*	7.73	47.61

Data are expressed as IC_{50} values (µg/mL) obtained from HaCaT cytotoxicity assays divided by corresponding MIC values (µg/mL) against each bacterial strain. Ratios (IC_{50}/MIC) indicate the selectivity index (SI), where higher values reflect a wider therapeutic window and more favorable safety margin. Interpretation: SI > 10 = favorable; SI = 1–10 = narrow; SI < 1 = unfavorable. MIC values were determined by broth microdilution. IC_{50} values were calculated from triplicate cytotoxicity assays (mean ± SEM)
